# Prospects of Band Structure Engineering in MXenes for Active Switching MXetronics: Computational Insights and Experimental Approaches

**DOI:** 10.3390/ma18010104

**Published:** 2024-12-30

**Authors:** Ganapathi Bharathi, Seongin Hong

**Affiliations:** 1 Department of Physics, Gachon University, Seongnam 13120, Republic of Korea; bharathigm@gmail.com; 2 Department of Semiconductor Engineering, Gachon University, Seongnam 13120, Republic of Korea

**Keywords:** semiconducting MXenes, electronic band structure, surface functionalization, strain engineering, double-transition metal MXenes

## Abstract

MXenes, two-dimensional (2D) transition metal carbides and nitrides, have shown promise in a variety of applications. The use of MXenes in active electronic devices is restricted to electrode materials due to their metallic nature. However, MXenes can be modified to be semiconducting and can be used for next-generation channel materials. The inherent metallic characteristics of pristine M_n+1_X_n_-structured MXene can be tuned to semiconducting by (i) functionalizing MXenes with different moieties, (ii) applying external strain, and (iii) varying the composition. These strategies effectively modify the metallic electronic structure of MXene into a semiconducting one. This review focuses on the potential of tuning the electronic band structure of MXenes by surface functionalization, strain engineering, and compositional variation. The computational and experimental approaches to tuning the electronic band structure using these strategies are discussed in detail. In addition, the experimental methods which can be used to prepare semiconducting MXenes are described.

## 1. Introduction

Silicon is the focus of modern electronics research. The miniaturization of solid-state electronics, as per Moore’s law projection, almost reached a saturation point in the 2000s owing to the scaling restrictions of existing silicon-based transistors [1,2]. Scientists have been extending the lifespan of Moore’s law yearly through supportive innovations such as metal gates with high-k dielectrics, fin field-effect transistor (FinFET) geometries, strain engineering, extreme ultraviolet photolithography, and device design engineering. Scaling down the transistor dimensions by reducing the dimensions of the silicon channels, typically to less than 5 nm, results in performance degradation due to short-channel effects (SCEs) [3,4]. In general, SCEs originate from the presence of dangling bonds and surface roughness in silicon when thinned. Although silicon satisfies all the fundamental requirements of an active switching channel and offers several advantages, including balanced electrical properties for a wide range of applications, scaling limitations have led scientists to explore alternatives beyond silicon.

When it was first discovered, graphene was considered a potential game-changer as a replacement for silicon in active electronic devices due to its exceptional properties, including high carrier mobility, excellent electrical conductivity, and remarkable mechanical strength [5,6]. However, its lack of a natural bandgap hindered its switching capabilities, limiting its use in active components [5,7,8]. Recent research has demonstrated various strategies to tune graphene’s electronic band structure and induce a bandgap, such as fabricating nanofragments (nanoribbons and quantum dots) and employing surface functionalization with groups like -OH, -O, and -NH_2_ [9,10]. While these approaches have successfully imparted semiconducting properties to graphene, they come at the cost of reduced carrier mobility and electrical conductivity, rendering it unsuitable as an active switching channel.

The transition metal dichalcogenides (TMDs) were later demonstrated to be promising alternatives to silicon and graphene [11,12]. TMDs can address the major drawbacks of silicon with their ability to perform at the atomic level and those of graphene with their natural bandgap. TMDs, especially MoS_2_, have been extensively examined as active channel materials and have demonstrated performances comparable to those of Si-based devices. However, achieving the large-scale uniformity and high contact resistance of metal electrodes remains challenging [13].

MXenes are an emerging class of two-dimensional (2D) materials that have gained significant attention for their unique combination of metallic conductivity and electronic structure tunability [14]. In general, these materials are derived from MAX phases, which are layered ternary carbides or nitrides. MXenes are produced by selectively etching out the ‘A’ element from the MAX phases, typically using hydrofluoric acid or other fluoride-containing solutions. This etching process results in 2D sheets of transition metal carbides, nitrides, or carbonitrides, denoted by the formula M_n+1_X_n_T_x_, where M represents an early transition metal, X stands for carbon or nitrogen, and T_x_ represents surface terminations such as -OH, -O, or -F [14,15]. One of the key advantages of MXenes lies in their inherent metallic conductivity, originating from the free electrons in the transition metal carbide/nitride backbone. This property is comparable to graphene, renowned for its exceptional electrical conductivity. However, unlike graphene, MXenes offer greater flexibility in tuning their electronic band structure without introducing defects into their core structure. The tunability of MXenes’ electronic structure originates from two primary factors—(i) surface functionalization: The surface of MXenes is typically terminated with functional groups, which significantly influence their electronic properties. The electronic band structure of MXenes can be tuned by surface functionalization with several groups, such as -OH, -O, -S, -Se, -Te, -F, -Cl, -Br, and -I, which in turn tune the bandgap between 0 and 3 eV [7,16,17]. (ii) Compositional variations: The choice of transition metals in the MXene structure also impacts its electronic properties. Different transition metals possess varying numbers of valence electrons, affecting the overall electronic configuration. Through the substitution of different transition metals, the bandgap can be tuned within a range of 0.24–0.96 eV [16]. For example, ordered double-transition metal MXenes can be engineered in which the outer transition metal layers play a more dominant role in determining the electronic properties than the inner core metals. The ability to precisely control the electronic properties of MXenes makes them highly versatile materials with potential applications in some key areas, including flexible electronics, energy storage, photonics, and optoelectronics.

**Figure 1:**
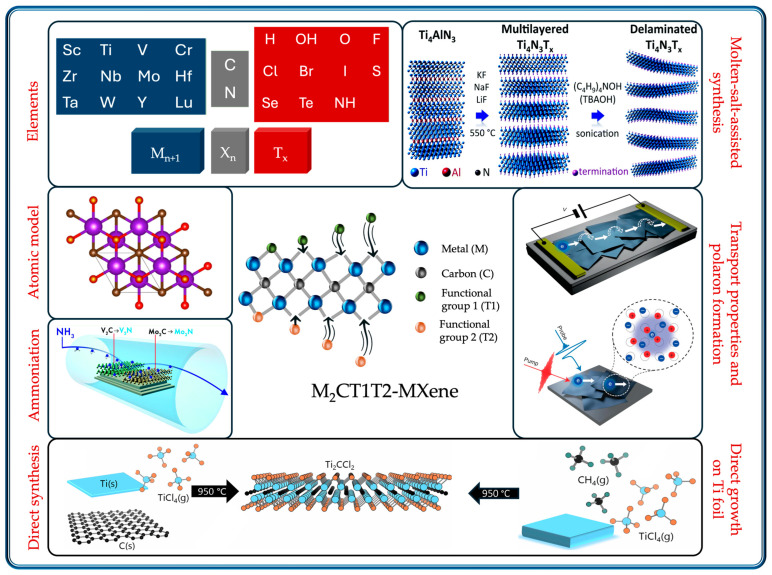
Representation of requirements for the successful creation of a semiconducting MXene channel material for a field-effect transistor application. Left panel—elements, atomic model (color code: Sc is pink, C is brown, O is red), and ammoniation process; bottom panel—direct synthesis and direct growth on Ti foil [18] (color code: Ti is blue, C is black, Cl is orange); right panel—molten-salt-assisted synthesis and transport property measurements of MXenes (charge transport represented by solid arrows is intra-flake and dashed arrows is inter-crystallite); middle panel—proposed MXene structure with different functionalization on top and bottom surfaces.

In this review, we discuss possible routes for the tuning of the electronic band structures of MXenes to convert them into semiconducting materials. First, the characteristics of an ideal switching channel are discussed, followed by the computational methods used to investigate the electronic properties of MXenes. Computational investigations of the electronic band structure of MXenes tuned by varying the surface termination, strain engineering, and composition are reviewed in detail. Experimental investigations of the characteristics of semiconducting MXenes, device performance, and transport properties are discussed. Experimental methods that can be used for the synthesis of semiconducting MXenes are described. Finally, concluding remarks and future research directions are discussed. Figure 1 illustrates the possible requirements for the successful creation of semiconducting MXenes.

## 2. Characteristics of an Ideal Switching Channel

Switching channels are at the heart of field-effect transistors and effectively determine the efficiency and speed of an electronic device. To achieve better performance, the switching channels must have certain characteristics, and semiconductor materials that satisfy these characteristics will be a good choice for the construction of electronic devices such as FETs, a Complementary Metal–Oxide–Semiconductor (CMOS), and other logical devices. The characteristics of an ideal switching channel are discussed below.

### 2.1. Bandgap

The bandgap is a crucial factor because it determines the ON/OFF ratio and the OFF-state leakage current. The bandgap of an ideal switching channel should be sufficiently large to suppress the OFF-state leakage current and ensure a high ON/OFF ratio. In general, a minimum bandgap of 0.4 eV is considered nominal for achieving a better ON/OFF ratio at room temperature [19]. However, when the bandgap increases, the carrier mobility decreases because they exhibit an inverse relationship. Therefore, the channel material should have an appropriate bandgap, and care should be taken when choosing it. The following equations explain the influence of the bandgap on the OFF current and ON/OFF ratios [20]:

(1) $$I_{OFF}\propto exp\frac{-E_{g}}{mk_{B}T}$$

(2) $$\frac{I_{ON}}{I_{OFF}}\propto exp\frac{E_{g}}{mk_{B}T}$$

where $E_{g}$ denotes bandgap and m is an ideality factor of 2 or larger, which depend on the specific FET design. The ideality factor m quantifies the deviation of a MOSFET’s subthreshold conduction from the ideal behavior. It indicates how effectively the gate voltage controls the channel current in the subthreshold region. $k_{B}$ denotes Boltzmann’s constant, and T denotes temperature.

### 2.2. Channel Thickness

Ultrathin channels are essential for better electrostatic gate control and for minimizing short-channel effects (SCEs). The effective control distance of the gate electric field extending into the channel is the scaling length (λ), which is proportional to the square root of the channel thickness. It is provided by the equation below [1,20]:

(3) $$\lambda =\sqrt{t_{ch}t_{bar}\varepsilon_{ch}/\varepsilon_{bar}}$$

where $t_{ch}$ and $t_{bar}$ denote thicknesses, and $\varepsilon_{ch}$ and $\varepsilon_{bar}$ denote the dielectric constants of the channel and the barrier gate, respectively.

(4) L≥a×λ

where a is the proportionality constant that establishes the minimum allowable channel length L, relative to the characteristic scaling length λ [21,22]. In practice, a often ranges from 2 to 3, indicating that the channel length should be at least 2 to 3 times the scaling length to ensure robust device performance.

The 2D channel materials, such as TMDs and MXenes, can be atomically thin and achieve sharper switching and a lower subthreshold swing.

### 2.3. High ON/OFF Ratio (I_ON_/I_OFF_)

For digital logical applications, a high ON/OFF ratio is required to ensure a clear distinction between the ON and OFF states. A FET device with a higher ratio indicates better switching efficiency and lower power consumption. It has been reported that an ON/OFF ratio of 10^4^–10^5^ is required for low-power operations, and a ratio of 1 × 10^4^–5 × 10^7^ is required for digital logic operations [20]. Silicon shows an optimal ON/OFF ratio; however, it degrades with a decrease in channel thickness beyond a certain limit, owing to the short-channel effect. Furthermore, 2D TMDs, such as MoS_2_, have demonstrated good ON/OFF ratios of up to 10^8^ [5,23]. The ON/OFF ratio is directly influenced by several factors such as the bandgap, effective mass, contact resistance, and interface quality.

### 2.4. Steep Subthreshold Swing (SS)

The subthreshold swing (SS) is the voltage required to change the drain current by an order of magnitude. The theoretical limit of the SS is 60 mV/dec at room temperature **[19]**. Achieving a steep SS requires a low interface charge density (ITC, =10^11^–10^13^ cm^−2^ eV) and minimal band tailing effects, which can be achieved by high-quality interfaces [24,25]. The SS is calculated using the following equation:

(5) $$SS={\left(\frac{\partial \log_{10}I_{DS}}{\partial V_{G}}\right)}^{-1}$$

where $I_{DS}$ denotes the source drain current, and $V_{G}$ denotes the gate voltage.

### 2.5. Low Leakage Current (I_OFF_)

The leakage current in the OFF state of the FET should be as low as possible, which is a requirement for low-power operation. The leakage current is determined by the bandgap of the channel material. It can be explained by Equation (6), which gives the relation between the leakage current, intrinsic carrier concentration, and bandgap of a material. For example, if the bandgap of a channel material is larger, its intrinsic carrier concentration is lower, and consequently, the leakage current is lower. Therefore, the larger the bandgap, the lower the leakage current. The expression given in Equation 6 denotes the relationship between the bandgap and the leakage current [20,25,26].

(6) $$I_{OFF}\propto n_{i}\propto exp\frac{-E_{g}}{mk_{B}T}$$

where $n_{i}$ denotes the intrinsic carrier concentration.

### 2.6. High ON-State Current

A high ON-state current is required to achieve fast switching speeds in FET devices. This is influenced by the carrier mobility, contact resistance, and effective mass of the channel material. Higher carrier mobility and lower contact resistance can result in a high ON-state current [26,27].

### 2.7. Temperature-Independent Subthreshold Swing (SS)

In an ideal case, the SS should remain steeper and unaffected by temperature variation, which indicates consistency in FET operation across different temperatures [28,29]. However, this remains a common challenge in FETs and requires the careful engineering of channel materials and device design.

### 2.8. Low Gate-to-Drain Capacitance (C_gd_)

The capacitance between the gate and the drain must be minimized to reduce power consumption and enhance switching speed [3]. Furthermore, the quantum capacitance (QC) originating from the finite density of states (DOS) of the channel material contributes to C_gd_ and can be controlled by choosing a channel material with a low DOS. Careful optimization of the device structure, gate-oxide thickness, and channel length can effectively decrease C_gd_.

### 2.9. Appropriate Effective Mass

Effective mass is a measure of how easily a charge carrier accelerates in response to an applied electric field. A low effective mass in the confinement direction can aid in reducing the DOS and gate-to-drain capacitance; alternatively, a high effective mass in the transport direction reduces source-to-drain tunnelling and aids in maintaining a good ON/OFF ratio [30,31].

### 2.10. High Carrier Mobility

Higher carrier mobility is essential to achieve a higher switching speed and low-power operation. Several factors affect carrier mobility; however, key factors, such as the choice of a channel material with high carrier mobilities and low channel thickness, can significantly aid in achieving better switching efficiencies [32,33].

### 2.11. Minimizing Short-Channel Effects (SCEs)

When the FET dimensions are scaled down, the channel length eventually decreases, and beyond a certain limit, the FET performance starts to degrade. This is technically a result of drain-induced barrier lowering (DIBL) and voltage roll-off effects induced by channel length shrinkage. The choice of ultrathin channel materials and high-quality interfaces can aid in addressing this issue [34,35].

### 2.12. Stable and Controllable Doping

Doping is an effective strategy for achieving desirable switching characteristics, including the modulation between n- and p-type conduction. Doping atomically thin 2D materials (especially TMDs) remains challenging because conventional doping strategies do not work as expected in the case of 2D TMDs [32].

### 2.13. Efficient Heat Dissipation

Effective heat dissipation is crucial to prevent performance degradation and ensure device reliability. Two-dimensional (2D) materials with high thermal conductivities offer potential advantages for better thermal management. Reducing the channel length and using materials such as h-BN as lateral heat spreaders can further enhance heat dissipation [30,31].

## 3. Computational Methodologies

Several computational methodologies have been used to examine the electronic band structures and bandgaps of semiconducting MXenes. Density functional theory (DFT) is a widely used method because of its reliability in predicting atomic-scale physical and chemical phenomena, although it is known to estimate bandgaps and van der Waals interactions with low accuracy [36,37]. The Perdew–Burke–Ernzerhof (PBE) version of generalized gradient approximation (GGA) has been widely used to study MXenes because of its balance between accuracy and computational costs, despite it providing less accurate bandgap values in certain cases [18,38]. Hybrid functionals, such as Heyd–Scuseria–Ernzerhof (HSE06), have been employed to investigate the electronic structure of MXenes, providing improved bandgap estimations with a more accurate representation of electronic properties [39,40,41,42].

The Boltzmann transport equation has been used to study the transport properties of MXenes such as their electrical conductivity [36,37,43]. This method is useful for correlating the electronic structures and transport characteristics of MXenes. A more in-depth analysis of the transport characteristics of MXenes can be performed using Rode’s iterative approach because it considers elastic and inelastic scattering in its calculations [37]. Furthermore, this method can provide insights into the type and behavior of charge carriers by determining the Hall scattering factor. The nonequilibrium Green’s function (NEGF) has been used to study the quantum transport properties of MXenes [44]. This method is powerful because it can be used to investigate electron transport in devices such as p-n junctions and device performance with respect to changes in doping, applied fields, and variable device geometries.

The structure and stability of MXenes can be investigated using phonon dispersion analysis, which examines the vibrational modes of atoms to provide insights into the dynamic stability of MXenes and their thermal properties [45,46]. Molecular dynamics simulations can also be used to study the dynamic stability of MXenes. Additionally, this method can be used to study structural changes in response to thermal fluctuations and other external factors [47].

## 4. Surface Functionalization

**Figure 2:**
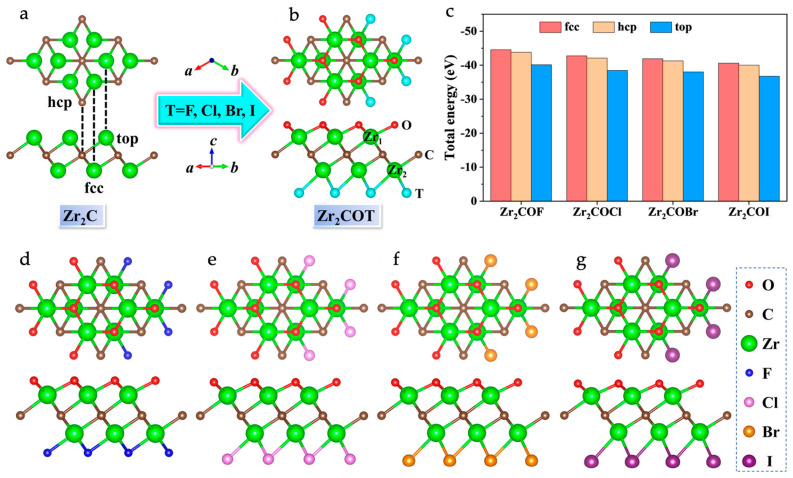
Top and side views of the (**a**) Zr_2_C monolayer and (**b**) Janus Zr_2_COT (T = F, Cl, Br, I) monolayer structures. (**c**) Energies of the three considered types of surface structures of Zr_2_COT monolayers. Top and side views of the Janus Zr_2_COT monolayers, showcasing (**d**) Zr_2_COF, (**e**) Zr_2_COCl, (**f**) Zr_2_COBr, and (**g**) Zr_2_COI. The labels “top”, “fcc”, and “hcp” indicate the positions of the functional groups in these configurations. Reproduced with permission from Ref. [48]. Copyright 2024, American Chemical Society.

MXenes are metallic or semi-metallic materials by nature. To render them semiconducting, it is necessary to perform structural changes such as surface functionalization, variation in composition, the application of strain, or a combination of these factors. Surface functionalization is a widely used method because it offers the tunability of electronic structures using a variety of functional groups. As an example, Figure 2 shows the atomic structure models of Zr-based MXenes with different functionalization [48]. During functionalization, the electronegative groups withdraw one or two electrons from transition metal atoms. This sharing of electrons results in changes in the electronic band structure, technically shifting the Fermi level from the valence d levels of transition metals, thereby leading to a reduction in the metallic nature of MXenes toward semiconducting behavior and bandgap opening.

Kzhazaei et al. investigated the electronic properties of a range of MXene systems, including M_2_C (where M represents Sc, Ti, V, Cr, Zr, Nb, or Ta) and M_2_N (where M represents Ti, Cr, or Zr), and modified the electronic structures of these MXenes by attaching F, OH, or O groups to their surfaces [36]. In this study, it was found that the Ti_2_C MXene became semiconducting with a downward shift in its Fermi level between those of Ti 3d and carbon p only when functionalized with O groups. This is because Ti has four valences (it can share up to two electrons). When F and OH groups were chosen for functionalization, they withdrew only one electron, causing the Fermi level of the MXene to shift, but not beyond the Ti 3d band. Similarly, Sc_2_C exhibited a transition to a semiconducting electronic structure upon functionalization with O and OH groups. The difference in the valences of the Ti and Sc metals resulted in the different tuning of the electronic structures for the same functional groups.

**Figure 3:**
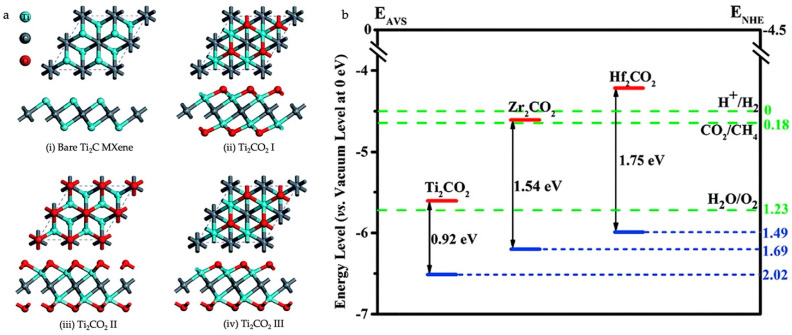
(**a**) Geometries of the (i) bare Ti_2_C MXene and the (ii, iii, and iv) Ti_2_C functionalized by the surface group of O with different geometries. (**b**) Band edge positions of the Ti_2_CO_2_, Zr_2_CO_2_, and Hf_2_CO_2_ (blue line represent the valence band and red line represent the conduction band). The redox potentials of H^+^/H_2_, H_2_O/O_2_, and CO_2_/CH_4_ at pH = 0 are also given as a reference (the green dashed lines). Reproduced with permission from Ref. [17]. Copyright 2016, Royal Society of Chemistry.

Xie et al. reported a semiconducting Ti_2_CO_2_ MXene with a bandgap of 0.88 eV calculated using the HSE06 functional, in which the functionalization of O groups on Ti_2_C layers opened the bandgap [42]. This study attributes the bandgap opening to the strong hybridization of Ti 3d and O 2p near the Fermi level, which in turn separates the valence and conduction bands, thereby leading to semiconducting behavior in the Ti_2_CO_2_ MXene. Koshi et al. reported the electronic band structures of Sc-based semiconducting MXenes Sc_2_CF_2_, Sc_2_CO_2_, and Sc_2_C(OH)_2_ with definite bandgap values [37]. The valence band minimum (VBM) was mostly contributed by the metal (Sc), whereas the conduction band minimum (CBM) was dominated by the functional groups (O and H). Zhang et al. reported the bandgap opening of M_2_CO_2_ MXenes (M = Ti, Zr, or Hf) via surface functionalization. The electronic interactions of the surface termination groups with the transition metal atoms of MXenes resulted in the formation of bandgaps [17]. The arrangement of functional groups on the MXene surface influences the electronic properties of MXenes. Among the three studied arrangements, geometry I is a favorable geometrical position for functionalization to achieve semiconducting MXenes, as shown in Figure 3. According to this study, p orbitals of the carbon atom contribute to the VBM, and the CBM mostly comprises M-d (M = Ti, Zr, and HF) states.

**Figure 4:**
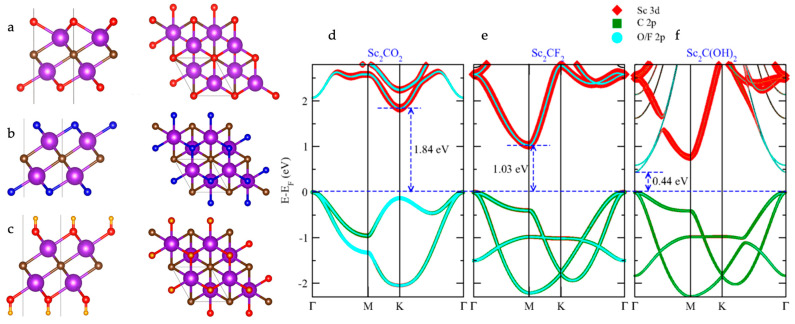
Atomic structure—side and top views of monolayer Sc_2_C functionalized by (**a**) O, (**b**) F, and (**c**) OH groups. Color code—Sc is pink, C is brown, O is red, F is blue, and H is yellow. Band structures with orbital weights of (**d**) Sc_2_CO_2_, (**e**) Sc_2_CF_2_, and (**f**) Sc_2_C(OH)_2_, respectively. Reproduced with permission from Ref. [43]. Copyright 2016, American Physical Society.

Hong et al. modelled armchair and zigzag nanoribbons and investigated their size- and edge-dependent electronic properties [49]. The width and atomic arrangement of MXene nanoribbons have a direct impact on the electronic band structure. For example, a Ti_2_CO_2_ 2D sheet with a bandgap of 0.32 eV displayed size-dependent bandgap behavior, which eventually increased for narrower nanoribbons. In contrast, an Sc_2_CO_2_ MXene demonstrated a decrease in the bandgap from 1.86 eV to 1.27 eV upon a decrease in size, which was attributed to the influence of strong edge states. He et al. reported the tuning of the electronic band structure of an Mn_2_CT_2_ MXene between the metallic, half-metallic, and semiconducting states based on the choice of surface termination [50]. Strong MnO interactions have been reported to result in the splitting of energy bands, which consequently opens the bandgap. Zha et al. reported the suitability of an Hf_2_CO_2_ MXene for nanoelectronic applications owing to their semiconducting electronic band structure [39]. The study suggests that the electronic band structure of Hf_2_CO_2_ is determined by the arrangement of Hf, C, and O atoms. We understand that O-group termination results in semiconducting-type electronic properties in Hf_2_CO_2_ material. In another study, Zha et al. reported that the strength of the interaction between the metal d-band and functional group p-band effectively determined the electronic band structure of semiconducting MXenes (Sc_2_CO_2_, Sc_2_CF_2_, Sc_2_C(OH)_2_, Mo_2_CF_2_, Ti_2_CO_2_, Zr_2_CO_2_, Hf_2_CO_2_, and W_2_CO_2_) [51]. Given that the MO interaction was much stronger when compared to other terminations, such as F and OH, the O-group-functionalized MXenes exhibited a semiconducting-type electronic band structure in most case studies. In another study, Kumar et al. reported a semiconducting-type electronic band structure in an Sc_2_C MXene functionalized with different groups, such as O, F, and OH groups [43]. Figure 4 shows the atomic structure of the functionalized Sc_2_C MXenes along with their electronic band structures. Variations in the electronic band structure and bandgap were observed depending on the electronegativity and consequent charge redistribution of these functional groups.

Si et al. reported the tuning of the electronic properties of Cr_2_C MXenes through surface functionalization with F, OH, H, or Cl groups [52]. Functionalization reportedly altered the electronic structure of Cr_2_C MXenes through the localization of d-electrons in Cr, the origin of semiconducting behavior, and bandgap opening in Cr_2_C MXenes. Yang et al. screened stable 2D Sc- and Y-based MXenes (M_2_CT_x_ (M = Sc and Y; T_x_ = O, OH, F, Cl, and Br)) among 2256 structures [7]. Tuning the surface functionalization with a range of functional groups resulted in bandgap tuning from 0.038 to 2.965 eV. It has been reported that the hybridization of functional groups with the d-band of transition metals creates new energy bands below the Fermi level, which causes the Fermi level to shift to the center of the bandgap between the transition metal d-band and the carbon p-band. This shift results in the transition of MXenes from metallic to semiconducting.

Bai et al. observed the semiconducting behavior of lutetium-based carbide (Lu_2_CT_2_) MXenes when the studied MXenes were functionalized with F and OH groups [53]. It was reported that the overlap of the 2p orbitals of the functional groups with the d and f orbitals of lutetium caused the splitting of d orbitals and the consequent formation of a bandgap. Novotny et al. investigated the effects of mixed terminations on the electronic properties and band structure of M_2_C MXenes, where M = Sc, Ti, or V. The combinations of OH, F, and O functional groups and the concentration of each group in the combination were systematically varied to study their detailed effects on the electronic structure and bandgap [54]. It is interesting to note that according to this report, an individual 2D MXene flake can be functionalized on both sides, and the electronic structure of the functional groups on each side does not influence the functional groups on the opposite side. This implies that the functional groups interact electronically only with the transition metal electronic bands and the C/N electronic bands on their sides. This study revealed a wide avenue for the fine-tuning of the electronic band structures of MXenes. Helmer et al. investigated the electronic properties of chalcogenide-functionalized MXene layers (M_2_CCh_2_, where M = Sc, Y, Ti, Hf, Zr, V, Nb, Ta, Mo, or W; and Ch = S, Se, or Te) [55]. Semiconducting behavior was observed in single-sheet MXenes (monolayers) and originated from the electronic interactions of the transition metal, carbon, and chalcogen functional groups. Mcrae et al. reported on Sc_2_C, a 2D semiconducting electride with a definite bandgap [56]. Electrides are a new class of materials that contain interstitial anionic electrons (IAEs), which occupy spaces between atoms rather than being associated with specific atoms. These interstitial electrons hybridize with electronegative metal cations and alter the band structure. When the metal is more electronegative, there is more hybridization, thereby opening up a definite bandgap. A list of MXenes with semiconducting properties due to band structure tuning by surface functionalization is presented in Table 1.

## 5. Strain Engineering

Strain engineering has been reported to be a useful strategy for tuning the electronic band structures of MXenes. In general, the applied strain causes changes in the lattice parameters, which in turn leads to a change in the electronic band structure of the corresponding MXenes. Although both tensile and compressive strains affect the electronic band structure, tensile strain mostly leads to the semiconducting characteristics of inherently metallic MXenes. Conversely, compressive strain can make MXenes more metallic in nature. Zha et al. reported the strain-induced tuning of the electronic band structure of an Hf_3_C_2_O_2_ MXene [40]. The application of strain causes a shift in the electronic bands and the selective opening of the bandgap. Upon the application of uniaxial/biaxial tensile strain, the overlap between the valence and conduction bands decreases, thereby resulting in a transition from a semi-metallic state to a semiconducting state. Alternatively, compressive strain increases the band overlap, which in turn results in the more metallic behavior of MXene. This type of anisotropic electrical conduction can be used in the construction of FET devices with the directional control of current flow.

Cui et al. examined the changes in the electronic band structure of semiconducting MXenes (Ti_2_CO_2_, Zr_2_CO_2_, Hf_2_CO_2_, Sc_2_CF_2_, Sc_2_C(OH)_2_, and Sc_2_CO_2_) upon the application of biaxial strain (compressive and tensile) [57]. The reported transition from an indirect to a direct bandgap in the examined MXene was due to the sensitivity of the localized states of the surface functional groups to the external strain. This study also revealed the possibility of continuously tuning the bandgap by applying various amounts of external strain. Sun et al. investigated the effect of biaxial strain on the electronic properties of a monolayer ZrTiCO_2_ double-transition metal MXene and observed a shift in the VBM and CBM positions, causing an increase in the bandgap value [58]. Ren et al. investigated the effect of biaxial strain (in the range of ±5%) on the electronic properties of a semiconducting yttrium-doped Sc_2_CO_2_ MXene [59]. The application of biaxial strain modified the lattice parameters of MXenes and influenced their electronic structure. Under tensile strain, there was a redshift in the CBM, which caused a reduction in the bandgap. Conversely, the application of a compressive strain of up to 1% caused an increase in the bandgap value, which then decreased owing to the redshift in the VBM.

Yue et al. tuned the electronic properties of Janus-structured MnCrNO_2_ MXenes by applying biaxial strain [60]. It was reported that when the strain was varied from –8% to +8%, the bandgap values of the studied MXene decreased significantly from 1.33 eV to 0.16 eV, and it became metallic for a strain value of +10%. Yan et al. examined the effect of biaxial strian ranging from –5% (compressive) to +5% (tensile) on the electronic properties of a semiconducting Cr-doped Sc_2_CO_2_ MXene [61]. A change in the bandgap was reported under different strain conditions (i.e., an increase in the bandgap from –5% to –3%, followed by a gradual decrease until +5%), which originated from the shift in the VBM in response to the applied strain. A list of semiconducting MXenes with band structures tuned by strain engineering is given in Table 2.

## 6. Composition and Structure

Double-transition metal and doped MXenes reportedly exhibit different electronic band structures from single-transition metal MXenes. The difference in the overall electronic structure originates from the electronic interactions of transition metals within and with cations/functional groups. This electronic interaction weakens the metallic characteristics of MXenes, which become semiconducting upon functionalization.

**Figure 5:**
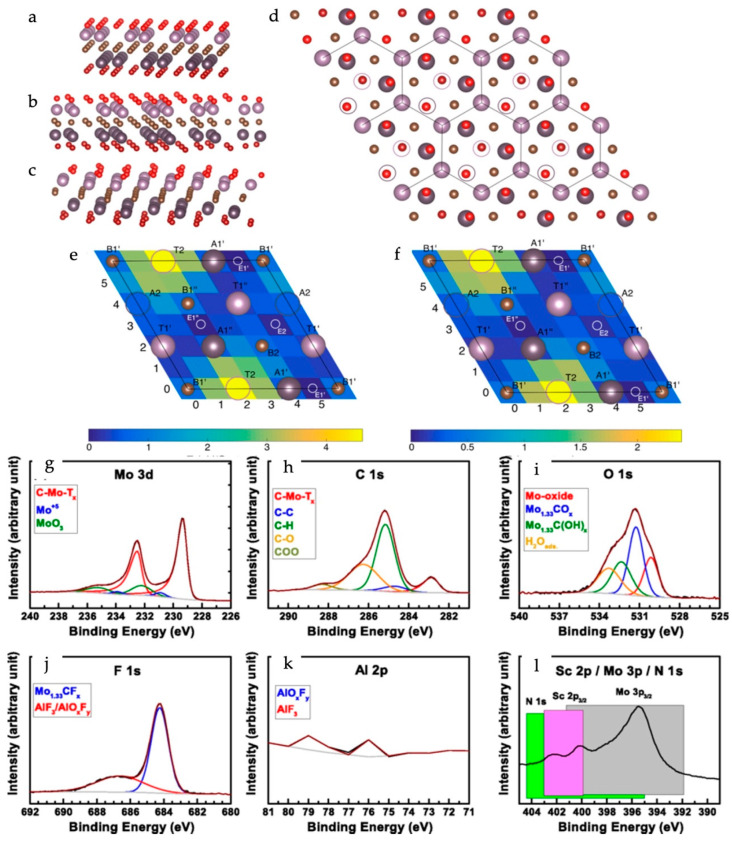
Schematic of termination positions in α-Mo_1.33_C, viewed from the (**a**) 010, (**b**) 110, and (**c**) 120 directions, and (**d**) from the top. The vacancies are marked with empty circles in (**d**). Color code: brown is C, purple is Mo, red is surface termination atoms, darker color represent the bottom layer. The energy surface representing the relative energies from placing (**e**) an O atom and (**f**) an F atom in the positions of a 6 × 6 grid across the Mo_1.33_C surface. XPS spectra with curve fitting of a Mo_1.33_CT_x_ freestanding delaminated film for (**g**) Mo 3d, (**h**) C 1s, (**i**) O 1s, (**j**) F 1s, (**k**) Al 2p, and (**l**) Sc 2p/Mo 3p. Various peaks represent various species assumed to exist. Labels and peak colors are coordinated. Reproduced with permission from Ref. [62]. Copyright 2016, American Physical Society.

Sun et al. reported the tuning of the electronic band structure of MXenes between metallic, semiconducting, and insulating by preparing them as double-transition metal MXenes [41]. In the studied TiM_2_X_2_T MXenes (where M = V, Cr, or Mn; X = C, or N; and T = H, F, O, or OH), it was demonstrated that replacing the surface metal and/or anion can result in a change in the electronic structure toward semiconducting behavior. For example, TiCr_2_N_2_ displays a metallic band structure, whereas TiCr_2_C_2_ exhibits semiconducting behavior. Additionally, the functionalization of these double-transition metal MXenes further opens avenues for the tuning of their electronic characteristics from metallic to semiconducting. For example, pristine TiCr_2_N_2_ and TiMn_2_N_2_ are metallic; however, when functionalized with -OH and -F terminations, the semiconducting TiCr_2_N_2_(OH)_2_ and TiMn_2_N_2_F_2_ exhibit a definite bandgap. Zhou et al. examined the electronic structural characteristics of double-layer and multilayer M_2_CO_2_ (M = Ti, Zr, or Hf) MXenes and reported the existence of inherent bandgaps in a few MXenes [44]. Changes in the electronic band structure and bandgap with respect to the number of MXene layers and the applied external electric field were investigated. It was determined that an increase in the number of MXene layers resulted in a slight decrease in the bandgap, which was due to increasing interlayer interactions. The application of an external electric field perpendicular to the MXene plane reportedly causes polarization and the consequent shifting of the CBM and/or VBM, thereby resulting in a decrease in the bandgap. Lind et al. investigated the electronic band structure of a vacancy-ordered Mo_1.33_C MXene [62]. Figure 5 shows the atomic structure of the functionalized Mo_1.33_C MXene along with the energy position mapping of the favorable binding sites for the functional group. The choice of functional groups played a key role in the tuning of the electronic structure to create a semiconducting MXene. Although the O-group functionalization of Mo_2_C (i.e., Mo_2_CO_2_) resulted in a metallic-type electronic structure, the Mo_1.33_C MXene faced stability issues upon O-group termination. The authors preferred F-group termination or an F-group-dominated F, O-group mixed termination for the tuning of the electronic band structure to achieve a semiconducting MXene while maintaining structural stability.

**Figure 6:**
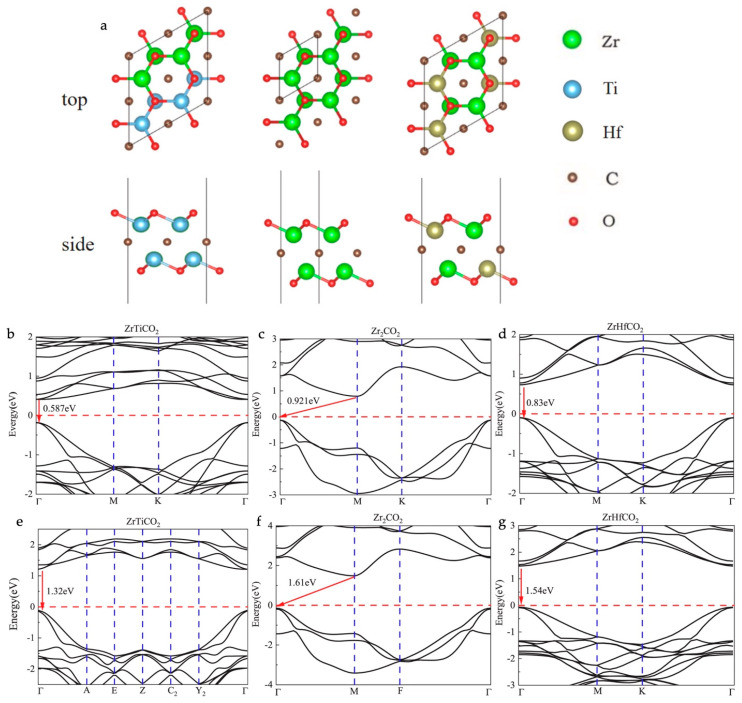
(**a**) Top and side views of the most stable configurations of ZrMCO_2_ (M = Ti, Zr, or Hf). Band structures of ZrMCO_2_ (M = Ti, Zr, or Hf) under PBE (**b**–**d**) and HSE06 methods (**e**–**g**). Reproduced with permission from Ref. [63]. Copyright 2016, Elsevier.

Zhang et al. explored changes in the electronic properties of ZrMCO_2_ MXenes (M = Ti or Hf) upon Janus structure formation by replacing one layer of Zr atoms with Ti or Hf atoms [63]. Primary ZrCO_2_ MXenes are semiconducting in nature, with an indirect bandgap value of 1.61 eV. The functionalized atomic structure models and their corresponding electronic band structures are presented in Figure 6. Upon the replacement of the Zr atoms with Ti and Hf atoms, a shift in the conduction band minimum occurred, resulting in reductions in the bandgap values to 1.32 and 1.54 eV, respectively. Furthermore, transition metal atom replacement causes a transition to a direct bandgap.

Bai et al. examined a range of lanthanide-based M_2_CT_2_ MXenes, where M = Ce, Pr, Nd, Sm, Eu, Gd, Tb, Dy, Ho, Er, Tm, or Yb [64]. Among all the metal atom–structure combinations examined, only the Gd_2_C MXene exhibited a semiconducting-type electronic band structure with F- and OH-group functionalization. Although the half-filled f orbital of the Gd atom was crucial for a favorable electronic band structure, the presence of F and OH functional groups further modified the electronic interactions, resulting in a bandgap opening.

The intrinsic semiconducting nature of Hf_3_C_2_O_2_ and Hf_2_CO_2_ MXenes was reported by Kumar et al., who stressed the influence of spin–orbit coupling on their electronic band structure [65]. This study revealed that the observed semiconducting-type electronic band structure was determined by the type of transition metal (Hf), the arrangement of carbon atoms, and surface functionalization with O groups. Wong et al. reported an intrinsic semiconducting-type electronic band structure in Ti_2(1−x)_Zr_2x_CO_2_, Ti_2(1−x)_Hf_2x_CO_2_, and Zr_2(1−x)_Hf_2x_CO_2_ MXene alloy systems, which were created by replacing the transition metals in Ti_2_CO_2_, Zr_2_CO_2_, and Hf_2_CO_2_ MXenes [66]. With good phase stability, these alloy systems formed disordered solid solutions in which the VBM was contributed by C and O p-orbitals, whereas the CBM was contributed by the transition metal d-orbitals.

Amoudeh et al. investigated the electronic properties of YMXT_x_ (M = Ti and Zr; X = C and N; T = H, O, and F) MXene solid solutions [67]. Without functionalization, all the MXene structures exhibited metallic characteristics. The electronic states around the Fermi level were influenced predominantly by the 4d orbitals of Y/Zr atoms and the 3d orbitals of Ti atoms, with a minimal contribution from the 2p orbitals of C/N atoms. After functionalization, YTiNH_2_ and YTiNF_2_ MXenes exhibited semiconducting properties. The 2p orbitals of the functional groups contributed to the density of states near the Fermi level and influenced the separation of electronic states. A list of semiconducting MXenes with band structures tuned by varying the composition and structure is presented in Table 3.

## 7. Experimental Investigations

Anasori et al. reported the preparation of semiconducting Mo_2_TiC_2_T_x_ and Mo_2_Ti_2_C_3_T_x_ MXenes, in which the origin of the semiconducting behavior was the replacement of Ti atoms with Mo atoms in Ti_3_C_2_/Ti_4_C_3_ MXenes [68]. The electronic interactions between the Ti and Mo atoms, along with the surface functionalization effects, contributed to bandgap formation.

Fitzgerald et al. reported a change in the metallic behavior of Nb_2_CT_x_ to semiconducting behavior when it was functionalized with N groups (i.e., Nb_2_CN_2_) [69]. This N-termination materialized through Nb-N bonding, leading to a shift in the Fermi level and consequent bandgap opening. In this case, surface functionalization with other groups (OH, –O, or –F) did not alter the electronic structure of the examined MXenes to a semiconductor. Hassan et al. reported the synthesis of a delaminated titanium nitride (d-Ti_4_N_3_T_x_) MXene using a modified oxygen-assisted molten-salt etching method [70]. The reported MXene exhibited semiconducting properties, which originated from the oxygen-group functionalization and replacement of oxygen in the nitrogen lattice (a partial oxynitride structure with O defects). A FET device was constructed using a d-Ti_4_N_3_T_x_ MXene as the active switching channel, which displayed p-type switching characteristics. It has also been claimed that a positive gate voltage contributes to an upward shift in the electronic bands, thereby leading to bandgap opening.

**Figure 7:**
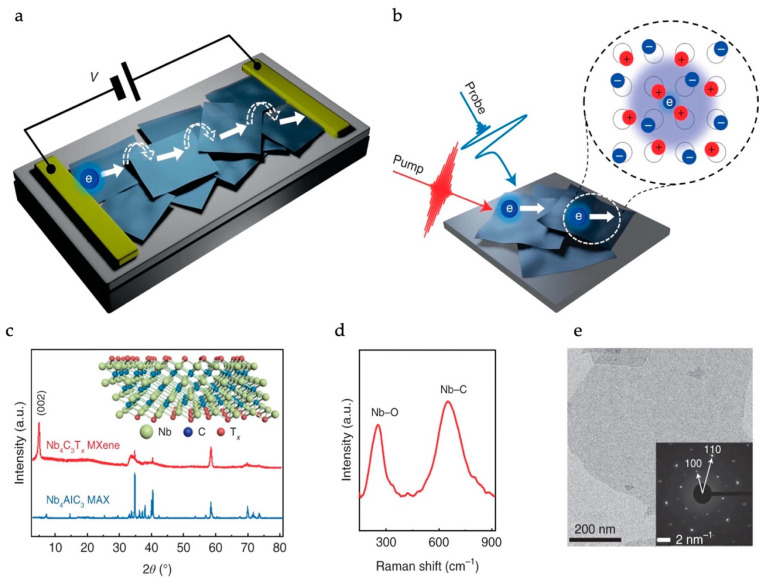
Charge transport measurement modes and characterizations of MXene samples. (**a**) Schematic of charge transport within the network of MXene flakes. Inter-crystallite charge carrier hopping (indicated by the dashed arrows) is revealed as the charge transport bottleneck, limiting the overall charge conduction between electrodes. (**b**) Scheme of ultrafast OPTP experiments to characterize the intra-MXene flake transport of charge carriers (indicated by the solid arrows). Inset: the formation of a large polaron, in which a conduction electron is dressed by lattice deformations extending over several lattice constants. (**c**) XRD spectra of Nb_4_AlC_3_ MAX (the mother compounds) and resultant Nb_4_C_3_T_x_ after etching. Inset: the crystal structure of the Nb_4_C_3_T_x_ MXene. (**d**) Raman spectrum of the delaminated Nb_4_C_3_T_x_ MXene. (**e**) TEM image of the Nb_4_C_3_T_x_ MXene. Inset: corresponding SAED pattern, which shows the high crystallinity and hexagonal symmetry of the crystal lattice. Reproduced with permission from Ref. [71]. Copyright 2022, Springer Nature.

Zheng et al. reported that the oxidation of Nb_4_C_3_T_x_ MXenes during an acid-assisted etching process results in an MXene structure with O-termination, which in turn exhibits a semiconducting electronic structure and a definite bandgap [71]. Intra- and inter-flake charge transport mechanisms in semiconducting Nb_4_C_3_T_x_ MXenes have been studied. A schematic of the charge transport within the network of MXene flakes is shown in Figure 7. Band-like transport occurs within individual flakes, and hopping transport is dominant for inter-flake charge transfer.

## 8. Synthesis Methods

The preparation of semiconducting MXenes relies heavily on the tuning of their functional groups and/or compositions. Therefore, care should be taken when preparing the MAX phases that yield a MXene after a series of chemical processes. In this section, we discuss the general procedures for preparing the MAX phases and the steps involved in MXene synthesis. In general, the MAX phases are ternary carbides and nitrides, denoted by the general formula M_n+1_AX_n_, where M denotes the transition metal, A is an A-group element, X is carbon and/or nitrogen, and n varies from one to nine [42,72,73,74,75]. It was recently reported that MXenes can be prepared without the parent MAX; however, the preparation of MXenes from the MAX phase has been widely performed. MAX-phase synthesis methods can be classified into four major categories, as described below.

### 8.1. Solid-State Reaction Methods

This protocol is widely used to prepare MAX phases. In this procedure, the precursor powders are mixed in desired stochiometric ratios, followed by heating at high temperatures in the range of 1100–1700 °C under an inert atmosphere [73,75,76]. Subclassifications of solid-state reaction methods with minor changes in the actual procedure have been reported, such as pressureless sintering, hot pressing (HP), spark plasma sintering (SPS), and self-propagating high-temperature synthesis (SHS) [77]. The solid-state reaction is versatile for the preparation of a wide range of MAX phases; however, it is time-consuming and yields undesirable products.

### 8.2. Physical Vapor Deposition (PVD)

This method has been used to prepare thin films of MAX phases by depositing the desired constituents on the target substrate in a controlled manner [73]. Methods such as Magnetron sputtering, cathodic arc deposition, and pulsed-laser deposition (PLD) fall into the PVD category. Furthermore, PVD is advantageous for creating high-purity coatings with better control over the composition; however, large-scale synthesis is a bottleneck.

### 8.3. Molten-Salt (MS) Synthesis

Molten-salt synthesis is a solid-state reaction; however, it differs because of its relatively low reaction temperature. Molten salts act as a medium to facilitate reactions and ion diffusion, which is favorable for the use of a wider range of precursors. The synthesis of nanostructured MAX phases, such as nanoflakes and nanofibers, can be achieved using 1D/2D carbon precursors [72,78]. It is important to note that preparing MAX phases with tailored morphologies can offer a better tunability of the structure and related properties of the resultant MXenes. Therefore, the molten-salt method is favorable for the synthesis of MXenes with selective structural modifications.

### 8.4. Microwave Synthesis

Microwave synthesis involves microwave heating for solid-state reactions. Given that the heating rate can be as high as 3000 °C per minute, the reaction durations are considerably reduced [76]. The microwave synthesis method can yield high-quality porous MAX phases with grain sizes in the range of 1–10 μm, which may require an additional milling step when compared to other synthesis methods.

## 9. MXene Synthesis

In general, MXenes are prepared by etching element A in the MAX phase, which is typically a ternary carbide and/or nitride, followed by delamination. Although the core structure of MXenes directly depends on the parent MAX phase, the etching, delamination, and post-etching treatment processes determine the structural quality through defect control, layer thickness, and surface functionalization. Therefore, it is important to carefully choose the etching and delamination methods to obtain the desired MXene structures.

### 9.1. Fluorine-Based Etching

**Figure 8:**
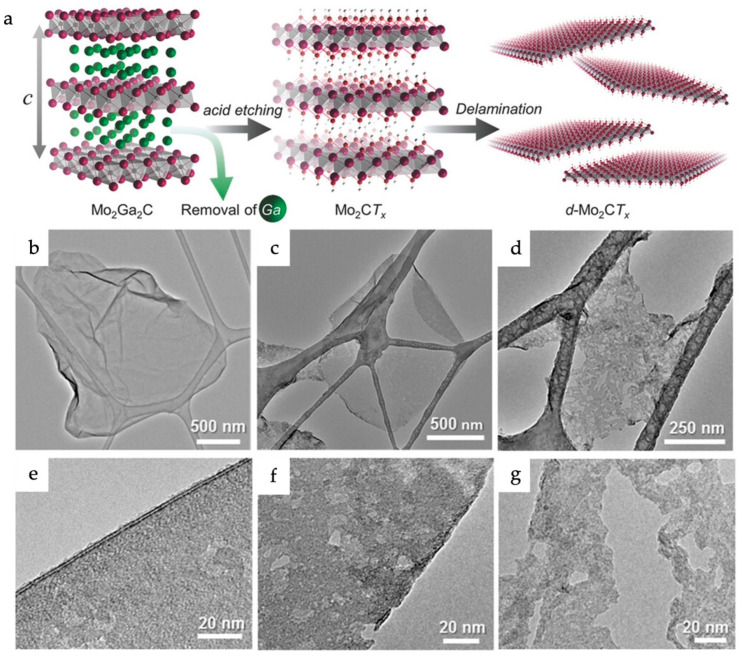
(**a**) Schematic showing synthesis and delamination of Mo_2_CT_x_ (color code: Pink is Mo, grey is C, green is Ga). Low-magnification TEM images of single flakes of (**b**) Mo_2_CT_x_–Li powders etched for 6 days, delaminated by manual shaking in water for 5 min, (**c**) Mo_2_CT_x_ after intercalation with TBAOH and hand shaking for 5 min and, (**d**) same as (**c**), but after sonication for 1 h in water. High-magnification TEM images of (**b**–**d**) are shown, respectively, in (**e**–**g**). Reproduced with permission from Ref. [79]. Copyright 2016, John Wiley & Sons.

Hydrofluoric acid (HF) is a conventional etchant used to etch element A. Fluorine has the highest chemical reactivity toward element A and can easily react and remove it from the MAX phase by forming an AF_3_ compound (A is aluminum in most cases) [15]. Despite its wide applicability, the highly corrosive nature and toxicity of HF remain a concern for the environment. To address this issue, researchers have developed an alternative in which a mixture of lithium fluoride (LiF) and hydrochloric acid (HCl) is used as the etchant. For example, an Mo_2_CT_x_ MXene was prepared by etching an Mo_2_Ga_2_C MAX phase using the LiF and HCl mixture (the schematic is shown in Figure 8) [79].Although fluorine was the etchant in this study, its in situ formation makes this method milder than the direct use of HF. Notably, while direct HF treatment produces MXenes with dominant fluorine surface terminations, the mixed etchant method allows control of the surface terminations. Recently, a refined version of this method, known as the Minimally Intensive Layer Delamination (MILD) technique, was reported, in which the etching process was performed at room temperature. Several other improvements to the MILD method, such as next-generation MILD (NGMILD) and Evaporated-Nitrogen MILD (EN-MILD), have also been reported, which can yield larger MXene flakes with minimal defects [80,81].

### 9.2. Molten-Salt Etching

Lewis acid molten salts can be an alternative to HF for the etching of MAX phases. For example, a ZnCl_2_ Lewis acid molten salt was used to etch Ti_3_AlC_2_ to yield Ti_3_C_2_Cl [72,82]. The actual etching process occurs in two steps: the molten salt reacts with the MAX phase to form an intermediate phase, which then reacts to produce the desired MXenes.

**Figure 9:**
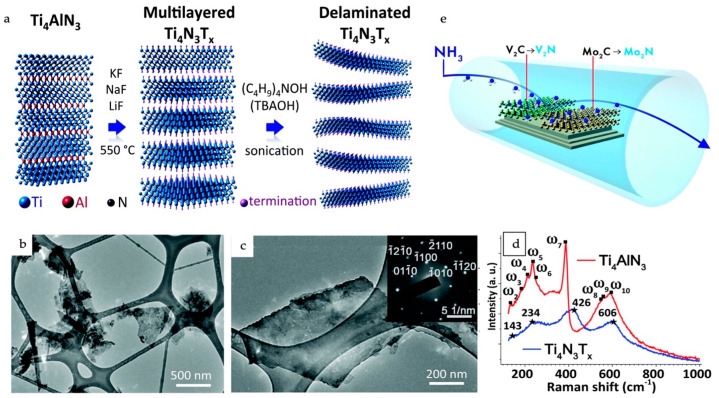
(**a**) Schematic illustration of the synthesis of Ti_4_N_3_T_x_ by the molten-salt treatment of Ti_4_AlN_3_ at 550 °C under Ar, followed by the delamination of the multilayered MXene by TBAOH. TEM micrographs of (**b**) several delaminated flakes and (**c**) an individual Ti_4_N_3_T_x_ flake. The inset in (**c**) shows the SAED pattern of the Ti_4_N_3_T_x_ flake showing the hexagonal basal plane symmetry of the parent MAX phase. (**d**) Raman spectra of Ti_4_AlN_3_ (red curve) and delaminated Ti_4_N_3_T_x_ (blue curve), with corresponding vibrational peaks found by fitting with a product of Gaussian and Lorentzian functions. Reproduced with permission from Ref. [83]. Copyright 2016, Royal Society of Chemistry. (**e**) Schematic representing the synthesis of 2D transition metal nitrides, which can be achieved by the ammoniation of carbide MXenes (Mo_2_CT_x_ and V_2_CT_x_) at elevated temperatures. Reproduced with permission from Ref. [85]. Copyright 2017, Royal Society of Chemistry.

Notably, the resultant MXenes have Cl groups at their surface terminations. In certain cases, the formation of new MAX phases, such as Ti_3_ZnC_2_, is also possible when conventional Al-based MAX phases react with the ZnCl_2_ molten salt. Similarly, nitride MXenes (Ti_4_N_3_T_x_) can also be prepared by the molten-salt treatment of Ti_4_AlN_3_ at 550 °C under argon (Ar), as illustrated in the schematic given in Figure 9 [83]. The preparation of nitride MXenes by the ammoniation of carbide MXenes has also been demonstrated [84].

A eutectic molten-salt mixture of alkali metal halides (LiCl and KCl) with a controlled amount of transition metal halides enabled the low-temperature etching (<750 °C) of MAX phases with the simultaneous delamination of the MXene layers. The alkali-etching-based synthesis of Ti_3_C_2_T_x_ was achieved by reacting Ti_3_AlC_2_ and NaOH in water, resulting in an F-group-free MXene structure [85]. The Bayer process aided in dissolving Al (oxide) hydroxides in NaOH at elevated temperatures and high NaOH concentrations.

### 9.3. Delamination

Delamination separates MXene layers with a desirable flake thickness. The etching of MAX phases yields MXenes with a large number of layers, which should be thinned down to use them in desirable applications. This delamination process involves intercalation followed by sonication. Intercalants, such as metal ions (e.g., Li, Na, and K+) and organic molecules, such as urea, dimethyl sulfoxide (DMSO), tetrabutylammonium hydroxide (TBAOH), and tetramethylammonium hydroxide (TMAOH), penetrate the MXene layers and weaken the interlayer forces [83,86,87,88]. These weakened MXene flakes delaminate into individual layers when subjected to gentle ultrasound treatment (i.e., the sonication process). One must be careful when choosing the sonication power and duration because an overdose can cause structural damage to the MXene layers.

MILD and molten-salt etching methods have been reported to delaminate MXene layers parallel to the etching process. It can be understood that the dissociated ion of the etchants used in these processes (particularly, Li ions) are capable of intercalating in the MXene layers, thereby resulting in parallel delamination.

### 9.4. Direct Synthesis

Recently, the bottom-up synthesis of MXenes without the use of the respective MAX phases has been reported, which is considered direct synthesis. Wang et al. reported the direct synthesis of Ti_2_CCl_2_ (DS-Ti_2_CCl_2_) using the ground powder of titanium and graphite with TiCl_4_, sealed in a quartz ampoule and heated at 950 °C [89]. A multigram-level yield of MXene powder was reported using a direct synthesis method. In parallel, a Ti_2_CCl_2_ MXene is directly grown on the Ti foil by the CVD method using a gas mixture of CH_4_ and TiCl_4_ at an elevated temperature of 950 °C. A comparative list of MXene synthesis methods is given in Table 4, which presents the synthesis steps, advantages, and challenges of each method.

## 10. Conclusions and Perspectives

Since they were first reported in 2011 by Naguib et al., 2D transition metal carbides and nitride MXenes have shown promise for a wide range of applications owing to their metallic and semi-metallic characteristics [15]. The lack of a natural bandgap limits their application as an electrode material in active electronics. Unlike graphene, MXenes offer a wide scope for the tuning of their electronic properties by varying surface functionalization and composition. Investigations of semiconducting MXenes with tuned electronic band structures can unlock their potential as active switching channels for next-generation electronic devices, termed MXetronics.

Several computational studies have been conducted on the electronic structures of MXenes with respect to changes in surface functionalization, compositional variation, and strain engineering. The Fermi level of metallic MXenes lies within the d-band of transition metals. This implies that owing to the positioning of the Fermi level within the d-bands of the transition metals, the corresponding MXenes remain metallic. To convert metallic-type MXenes into semiconducting materials, the Fermi level should be pushed downward to the position between the d-bands of the transition metals and p-bands of carbon and nitrogen. This change in electronic band structure can be realized by a few external changes, such as the modification of surface functionalization and composition, and strain engineering.

Surface functionalization is an efficient strategy for the tuning of the band structure of MXenes to introduce semiconducting behaviors. The advantage of MXenes lies in the range of options that allow the functional groups to attach to the open ends of transition metals, which include -OH, -O, -S, -Se, -Te, -F, -Cl, -Br, and -I groups. When these functional groups are attached to the transition metal edges of the MXens, they withdraw electrons from the transition metals, thereby pushing the Fermi level downward. Additionally, the strong hybridization of the transition metal d-orbitals with the p-orbitals of the functional groups creates a definite bandgap in functionalized MXenes. The electronegativity of the functional groups determines their interaction strength with the transition metal atoms and consequently, the band structure variation. For example, the F groups are less electronegative, withdrawing only one electron from one metal atom of an MXene, and cannot convert the corresponding MXene into a semiconducting material. Alternatively, O groups withdraw two electrons from the transition metal atoms and turn the MXene into a semiconductor in most cases.

Strain engineering in MXenes provides another avenue for the tuning of electronic properties. The application of tensile strain (uniaxial and biaxial) decreases the overlap between the conduction and valence bands, leading to a bandgap opening and consequent conversion to a semiconducting state. Conversely, compressive strain increases the overlap of the conduction and valence bands, thereby switching to metallic characteristics. Compositional variation is another route to tune the electronic properties of MXenes, which can be achieved through transition metal substitutions. An example of a double-transition metal MXene, TiM_2_X_2_T_x_ (M = V, Cr, or Mn; X =C or N; T = H, F, O, or OH), demonstrates a wide range of electronic behaviors, from metallic to semiconducting, depending on the metal and anion combinations. Compositional variations extend the tunability of the electronic structure of MXenes.

Notably, surface functionalization is required for both double-transition metals and strain-engineered MXenes to induce semiconducting behavior, which indicates the overall importance of surface functionalization. Gaining complete control over the synthesis of MXenes is difficult; however, obtaining semiconducting MXenes is inevitable. The molten-salt etching method is favorable, particularly for eutectic mixtures of alkali metal halides and transition metal halides. This process can yield MXenes with the desired functionalization with minimal processing steps and lower reaction temperatures. Alternatively, PVD methods can yield MXenes with a higher electronic quality.

Future research can be conducted in the following directions: (i) the functional group-selective synthesis of MXenes with tunable electronic properties, (ii) the synthesis and functionalization of high-quality MXenes through PVD methods, (iii) the synthesis of new double-transition metal MXenes with semiconducting electronic characteristics, (iv) tuning the conductivity of MXenes for application in specific electronic devices, and (v) an investigation of inter-flake and intra-flake transport properties in different semiconducting MXenes.

## Figures and Tables

**Table 1:** List of semiconducting MXenes with band structures tuned by surface functionalization and their corresponding surface termination, bandgap values, bandgap value calculation method, and references.

MXene	Termination	Bandgap (eV)	Bandgap Calculation Method	References
Sc_2_C	OH	1.092, Direct	HSE06	[7]
	O	2.965, Indirect		
	F	1.939, Indirect		
	Cl	1.756, Indirect		
	Br	1.587, Indirect		
	I	0.864, Indirect		
	S	2.504, Indirect		
	Se	2.051, Indirect		
Y_2_C	OH	1.171, Direct	HSE06	[7]
	O	2.369, Indirect		
	F	1.922, Indirect		
	Cl	1.688, Indirect		
	Br	1.630, Indirect		
	I	1.205, Indirect		
	S	1.317, Indirect		
	Se	0.923, Indirect		
Mn_2_C	O	0.587, Indirect	HSE06	[7]
Zr_2_C	O	1.612, Indirect	HSE06	[7]
Mo_2_C	F	0.846, Indirect	HSE06	[7]
	Cl	0.623, Indirect		
	Br	0.230, Indirect		
Hf_2_C	O	1.831, Indirect	HSE06	[7]
V_2_C	S	0.201, Direct	HSE06	[7]
	Se	0.186, Indirect		
Nb_2_C	S	0.219, Direct	HSE06	[7]
	Se	0.382, Indirect		
Ru_2_C	OH	1.099, Indirect	HSE06	[7]
	F	1.978, Indirect		
	Cl	1.825, Indirect		
	Br	1.412, Indirect		
Ta_2_C	S	0.370, Direct	HSE06	[7]
	Se	0.387, Indirect		
Ti_2_C	O	0.741, Indirect	HSE06	[7]
Pd_2_C	O	0.407, Indirect	HSE06	[7]
W_2_C	Cl	0.450, Indirect	HSE06	[7]
Pt_2_C	O	0.744, Indirect	HSE06	[7]
Sc_2_C	OH	0.45, Indirect	DFT GGA-PBE	[36]
	O	1.8, Indirect		
	F	1.03, Indirect		
Ti_2_C	O	0.24, Indirect	DFT GGA-PBE	[36]
Zr_2_C	O	0.88, Indirect	DFT GGA-PBE	[36]
Hf_2_C	O	1.0, Indirect	DFT GGA-PBE	[36]
Sc_2_C	OH	0.34, Direct	DFT GGA-PBE	[37]
	O	1.83, Indirect		
	F	0.97, Indirect		
Ti_2_C	O	0.92, Indirect	HSE06	[17]
Zr_2_C	O	1.54, Indirect	HSE06	[17]
	F	0.25, Direct		
Hf_2_C	O	1.75, Indirect	HSE06	[17]
	F	0.42, Direct		
Ti_2_C	O	1.021	GGA-PBE	[39]
		1.657, Indirect	HSE06	
Ti_2_C	O	0.24	GGA-PBE	[42]
		0.88	HSE06	
Sc_2_C	OH	0.44, Direct	GGA	[43]
	O	1.84, Indirect		
	F	1.03, Indirect		
Ti _2_C	O	0.32, Indirect	PBE	[49]
Zr _2_C	O	0.97, Indirect	PBE	[49]
Hf _2_C	O	1.03, Indirect	PBE	[49]
Sc_2_C	OH	0.56, Direct	PBE	[49]
	O	1.86, Indirect		
	F	1.03, Indirect		
Y_2_C	OH	0.47, Direct	PBE	[49]
	O	1.32, Indirect		
	F	1.14, Indirect		
La_2_C	OH	0.64, Indirect	PBE	[49]
	O	0.60, Indirect		
	F	1.02, Indirect		
Hf_2_C	O	1.03, Indirect	PBE	[49]
Sc_2_C	O	1.78	GGA-PBE	[51]
		2.87, Indirect	HSE06	
	F	1.01	GGA-PBE	[51]
		1.85, Indirect	HSE06	
	OH	0.445	GGA-PBE	[51]
		0.845, Direct	HSE06	
Mo_2_C	F	0.301	GGA-PBE	[51]
		0.858, Indirect	HSE06	
Ti_2_C	O	0.261	GGA-PBE	[51]
		0.917, Indirect	HSE06	
Zr_2_C	O	0.966	GGA-PBE	[51]
		1.70, Indirect	HSE06	
Hf_2_C	O	1.02	GGA-PBE	[51]
		1.66, Indirect	HSE06	
W_2_C	O	Metallic	GGA-PBE	[51]
		0.0683, Indirect	HSE06	
Cr_2_C	OH	1.43, Indirect	HSE screened Coulombic hybrid density functional	[52]
	F	3.49, Indirect		
	H	1.76, Direct		
	Cl	2.56, Indirect		
Lu_2_C	OH	1.28, Direct	HSE06	[53]
	F	2.07, Indirect		
Ti_2_C	O	0.758, Direct	meta-GGA SCAN GGA-PBE	[54]
		0.543, Indirect		
Sc_2_C	F2c(OH)2(1 − c) (0 ≤ c ≤ 1)	0.499–1.507, Direct	meta-GGA SCAN	[54]
		0.499–1.061, Indirect	GGA-PBE	
Nb_2_C	S	0.21	optB86b-vdW-DF	[55]
		0.1	mBJ	
	Se	0.32	optB86b-vdW-DF	[55]
		0.30	mBJ	
	Te	0.06	optB86b-vdW-DF	[55]
		0.06	mBJ	
Ta_2_C	S	0.28	optB86b-vdW-DF	[55]
		0.17	mBJ	
	Se	0.34	optB86b-vdW-DF	[55]
		0.31	mBJ	
V_2_C	Se	0.19	optB86b-vdW-DF	[55]
		0.12	mBJ	

GGA-PBE—Perdew–Burke–Ernzerhof (PBE) version of generalized gradient approximation (GGA); HSE—Heyd–Scuseria–Ernzerhof; mBJ—modified Becke–Johnsson; optB86b-vdW-DF—van der Waals density functional (vdW-DF) with optB86b exchange.

**Table 2:** List of semiconducting MXenes with band structures tuned by strain engineering and their corresponding surface termination, strain values, bandgap values, bandgap value calculation method, and references.

MXene	Termination	Strain	Bandgap (eV)	Bandgap Calculation Method	References
Hf_3_C_2_	O	Biaxial −0.05 to 0.05	~0.0 to 0.555	HSE06	[40]
	O	Uniaxial along × axis −0.05 to 0.05	~0.0 to 0.338	HSE06	[40]
	O	Uniaxial along × axis −0.05 to 0.05	~0.0 to 0.189	HSE06	[40]
Sc_2_C	O	Biaxial −4 to 6%	~1.25 to 2.0	DFT+U and HSE06	[57]
	F	Biaxial −4 to 6%	~0.5 to 1.25	DFT+U and HSE06	[57]
	OH	Biaxial −4 to 6%	~0.0 to 0.75	DFT+U and HSE06	[57]
Ti_2_C	O	Biaxial −4 to 6%	~0.0 to 0.5	DFT+U and HSE06	[57]
Zr_2_C	O	Biaxial −4 to 6%	~0.5 to 1.25	DFT+U and HSE06	[57]
Hf_2_C	O	Biaxial −4 to 6%	~0.5 to 1.25	DFT+U and HSE06	[57]
ZrTiC	O	Biaxial 0 to 2%	~0.6773 to 0.7378	GGA-PBE	[58]
MnCrN	O	Biaxial −10 to 10%	~0.35 to 1.75	HSE06	[60]
	O	Biaxial −10 to 10%	~0.0 to 1.4	GGA-PBE	[60]
Sc_2_C-Y	O	Biaxial −5 to 5%	~1.4 to 2.0	GGA-PBE	[59]
Sc_2_C-Cr	O	Biaxial −5 to 5%	~0.1 to 1.0 (spin-up) ~1.2 to 2.0 (spin-down)	GGA-PBE	[61]

**Table 3:** List of semiconducting MXenes with band structures tuned by varying composition and structure, and their corresponding surface termination, bandgap values, bandgap value calculation method, and references.

MXene	Termination	Bandgap (eV)	Method	References
Hf_3_C_2_	O	0.43, Indirect 0.71, Direct	PBE	[65]
	O	1.08, Indirect 1.42, Direct	GW	[65]
Hf_2_C	O	0.99, Indirect 2.12, Direct	PBE	[65]
	O	2.18, Indirect 3.48, Direct	GW	[65]
TiCr_2_C_2_	Bare	0.1, Indirect	DFT+U	[41]
	Bare	0.4, Indirect	HSE06	[41]
	H	0.2, Indirect	DFT+U	[41]
	H	0.5, Indirect	HSE06	[41]
	O	0.1, Indirect	DFT+U	[41]
	O	0.4, Indirect	HSE06	[41]
	F	0,6, Indirect	DFT+U	[41]
	F	1.0, Indirect	HSE06	[41]
	OH	1.3, Indirect	DFT+U	[41]
	OH	1.9, Indirect	HSE06	[41]
TiCr_2_N_2_	O	0.2, Indirect	DFT+U	[41]
	O	0.6, Indirect	HSE06	[41]
	OH	0.5, Indirect	DFT+U	[41]
	OH	0.8, Indirect	HSE06	[41]
TiMn_2_N_2_	H	0.1, Indirect	DFT+U	[41]
	F	1.2, Indirect	DFT+U	[41]
	F	1.9, Indirect	HSE06	[41]
	O	0.3, Indirect	DFT+U	[41]
Ti_2_C (monolayer)	O	0.32, Indirect	GGA-PBE	[44]
Ti_2_C (double layer)	O	0.039, Indirect	GGA-PBE	[44]
Zr_2_C (monolayer)	O	0.97, Indirect	GGA-PBE	[44]
Zr_2_C (doube layer)	O	0.729, Indirect	GGA-PBE	[44]
Zr_2_C (multilayer)	O	0.597, Indirect	GGA-PBE	[44]
Hf_2_C (monolayer)	O	1.03, Indirect	GGA-PBE	[44]
Hf_2_C (double layer)	O	0.824, Indirect	GGA-PBE	[44]
Hf_2_C (multilayer)	O	0.724, Indirect	GGA-PBE	[44]
ZrTiC	O	0.79, Direct	GGA-PBE	[63]
	O	1.32, Direct	HSE06	[63]
Zr_2_C	O	0.921, Indirect	GGA-PBE	[63]
	O	1.61, Indirect	HSE06	[63]
ZrHfC	O	0.85, Direct	GGA-PBE	[63]
	O	1.54, Direct	HSE06	[63]
Gd_2_C	F	0.732, Indirect	GGA-PBE	[64]
	F	1.38, Indirect	HSE06	[64]
	OH	0.4, Direct	GGA-PBE	[64]
	OH	0.882, Direct	HSE06	[64]
Hf_2_MnC_2_	O	0.238, Indirect	DFT+U	[46]
	F	1.027, Indirect	DFT+U	[46]
Hf_2_VC_2_	O	0.055, Indirect	DFT+U	[46]
Ti_2(1−x)_Zr_2x_C	O	~1.2 to 1.8, Indirect	HSE06	[66]
Ti_2(1−x)_Hf_2x_C	O	~1.2 to 1.9, Indirect	HSE06	[66]
Zr_2(1−x)_Hf_2x_C	O	~1.7 to 1.8, Indirect	HSE06	[66]
YTiN	H	1.1	HSE06	[67]
YTiN	F	2.3	HSE06	[67]

**Table 4:** List of MXene synthesis methods with synthesis steps, synthesized MXenes, termination, advantages, challenges, and references.

Mxene Synthesis Method	Synthesis Steps	MXene and Termination	Advantages	Challenges	References
HF etching	HF used as etchant; Etching at 55 °C for 6.6 days; Intercalation with TBAOH; Delamination by mild sonication or hand shaking	Mo_2_CT_x_ T = OH, O, F	Scalability; Control over flake morphology	Termination control; Defect formation; TBAOH and water removal	[79]
Minimally Intensive Layer Delamination (MILD)	LiF-HCl mixture used as etchant; Etching at 35 °C for 6–16 days; Delamination by mild sonication or hand shaking	Mo_2_CT_x_ T = OH, O, F	Control over flake morphology; Scalability	Longer synthesis duration; Defect formation	[79]
Next-generation Minimally Intensive Layer Delamination (NG-MILD)	Mixture of HCl, LiF, and NH_4_Cl used as etchant; Etching at 60 °C for 120 h; Post-synthesis H2SO4 treatment; Delamination by sonication	Ti_3_C_2_T_x_, T = OH, O, F, Cl	Better colloidal stability; Improved etching efficiency; Scalability; Improved purity	Undesirable MXene sheet fragmentation; Removal of Li_3_AlF_6_ impurity	[80]
Evaporated-Nitrogen Minimally Intensive Layer Delamination (EN-MILD)	LiF-HCl mixture used as etchant; Etching at 35 °C for up to 30 h with continuous dry-N_2_-purging; Delamination by sonication and/or hand shaking	Ti_3_C_2_T_x_, T = OH, O, F	Exceptional electrical conductivity; High synthesis yield up to 60% after delamination; High colloidal concentration with better dispersibility	Sensitivity to process parameters such as nitrogen flow rate; Potential for defect formation at longer etching times;	[81]
Molten-salt etching	Eutectic mixture of Lewis acidic metal halide salts used as etchant; Etching at elevated temperatures ~200 to 750 °C	Multiple	Possible to achieve halide termination; Fluorine-free synthesis; Scalability	High-temperature requirement; In situ formation of metal nanoparticles; Removal of metal intercalants; Limited understanding of defect formation	[72]
	Mixture of KF, LiF, and NaF used as etchant; Etching at 550 °C for 30 min; Intercalation with TBAOH; Delamination by probe sonication	Ti_3_N_2_T_x_, T = OH, O, F	Possible synthesis of nitride; MXene scalability;	High-temperature requirement; Removal of metal intercalants	[84]
Alkali-assisted hydrothermal method	Alkali-assisted hydrothermal treatment under argon atmosphere at 270 °C; Intercalation with DMSO; Delamination by sonication	Ti_3_C_2_T_x_, T = OH, O	Fluorine-free synthesis; Non-fluorine termination; High-purity ~92 wt%	Highly sensitive to temperature and NaOH concentration; Appreciable surface oxidation into TiO_2_	[85]
CVD growth	Titanium or zirconium foil exposed to gas mixture of metal halide, CH_4_ or N_2_, and Ar at 600 to 1000 °C, for 15 min	Ti_2_CCl_2_, Ti_2_NCl_2_, Zr_2_CCl_2_, Zr_2_CBr_2_	Potential for patterned growth; Direct synthesis of new phases; Control over termination	Requirement of sophisticated tools	[89]
Direct synthesis	Solid-state reaction using titanium metal (Ti), graphite, and TiCl_4_ at 950 °C for 2 h to 10 days; Intercalation with n-butyllithium in hexane; Delamination by shaking	Ti_2_CCl_2_	Fluorine-free synthesis; Control over termination	Requirement of sophisticated tools; High reaction temperatures; Removal of byproducts	[89]

In a few cases, the advantages and drawbacks are not specifically mentioned in the corresponding references.
